# Supplementary material for: Marker-Assisted Selection in Breeding for Fruit Trait Improvement: A Review
Source: Int J Mol Sci. 2023 May 19;24(10):8984. doi: 10.3390/ijms24108984 (PMC10219056; doi:10.3390/ijms24108984)
Supplement: Supplementary file 1 [file ijms-24-08984-s001.zip › ijms-2398153-supplementary.pdf]

Supplementary Table S1. List of the most relevant references on MAS breeding

Character	Species	References	DOI
Phenological traits related to fruit production	Apricot	Campoy et al., 2011	10.1007/s11105-010-0242-9
	Peach	Dirlewanger et al., 2006	10.1007/s11295-006-0053-1
	Kaki	Akagi et al., 2014	10.2503/jjshs1.CH-109
	Ginco	Liao et al., 2009	10.1007/S10681-009-9913-8
	Kiwifruit	De Mori et al., 2022	10.3233/JBR-211530
	Mulberry	Atsumi et al., 2019	10.1007/S10681-019-2511-5
	Papaya	Vashistha et al., 2016	10.5376/MPB.2016.07.0028
	Pistacio	Şahin et al., 2022	10.1007/s11033-022-07285-5
Skin color	Apple	Moriya et al., 2017	10.1007/s10681-017-1864-x
	Apple	Chagne et al., 2016	10.1007/s11295-016-1025-8
	Peach	Breto et al., 2017	10.1007/s10681-016-1812-1
	Strawberry	Labadie et al., 2022	10.3389/fpls.2022.869655
Flesh color	Cherry	Stegmeir et al., 2015	10.1007/s11032-015-0337-y
	Papaya	Blas et al., 2010	10.1104/pp.109.152298
	Peach	Adamy et al., 2013	10.1007/s11105-013-0628-6
Fruit size and weight	Sweet cherry	De Franceschi et al., 2013	10.1007/s11032-013-9872-6
	Grapevine	Ban et al., 2016	10.1007/s10681-016-1737-8
	Japanese plum	Salazar et al., 2020	10.1016/j.postharvbio.2020.111292
Seedlessness	Citrus	Chavez et al., 2011	10.21273/HORTSCI.46.5.693
	Citrus	JinPing et al., 2009	10.1016/j.scienta.2009.03.006
	Grapevine	Wang 1997	
	Grapevine	Lahogue et al. 1998	10.1007/s001220050976
	Grapevine	Mejía and Hinrichsen 2003	10.17660/ActaHortic.2003.603.74
	Grapevine	Cabezas et al., 2006	10.1139/g06-122
	Grapevine	Royo et al., 2018	10.1104/pp.18.00259
	Grapevine	Wang et al., 2022	10.1016/j.jia.2022.07.047
Firmness, crispness, texture	Apple	Costa et al., 2010	10.1093/jxb/erq130
	Apple	Migicovsky et al., 2021	10.3389/fgene.2021.671300
	Apple	Longhi et al., 2013	10.1007/s11032-013-9912-2
	Grapevine	Ban et al., 2016	10.1007/s10681-016-1737-8
	Sweet cherry	Font i Forcada et al., 2013	10.1007/s11295-012-0553-0
Pulp acidity and pH	Apple	Chagne et al., 2019	10.1038/s41438-018-0114-2
	Apricot	Salazar et al., 2013	10.1007/s11105-013-0625-9
	Cranberry	Fong et al., 2020	10.1007/s11295-020-01432-4
	Cranberry	Fong et al., 2021	10.1007/s11295-020-01482-8
	Grapevine	Ban et al., 2016	10.1007/s10681-016-1737-8
	Peach	Wang et al., 2016	10.1007/s10681-016-1709-z
	Peach	Eduardo et al., 2014	10.1007/s11295-014-0789-y
VOCs	Strawberry	Noh et al., 2017	10.3233/JBR-160145
Harvesting date, maturity date, ethylene, ripening	Apple	Larsen et al., 2019	10.3835/plantgenome2018.12.0104
	Peach	Nunez-Lillo et al., 2015	10.1007/s11295-015-0911-9
	Raspberry	Graham et al., 2009	10.1007/s00122-009-0969-6
